# Association of Prenatal Depression With Second to Fourth Digit Ratio in Children Aged 4 and 6 Years

**DOI:** 10.1155/da/6655082

**Published:** 2025-05-26

**Authors:** Ou Zhang, Yao Chen, Yafei Chen, Ziliang Wang, Qian Sun, Hong Liang, Wei Yuan, Wei Sheng, Maohua Miao

**Affiliations:** ^1^Department of Nosocomial Infection Administration, Affiliated Jiangmen TCM Hospital of Jinan University, Jiangmen 529031, Guangdong, China; ^2^Shanghai-MOST Key Laboratory of Health and Disease Genomics, NHC Key Laboratory of Reproduction Regulation, Shanghai Institute for Biomedical and Pharmaceutical Technologies, School of Public Health, Fudan University, Shanghai 200237, China; ^3^Department of Nephrology, Zhongshan Hospital, Fudan University, Shanghai, China; ^4^Shanghai-MOST Key Laboratory of Health and Disease Genomics, NHC Key Laboratory of Reproduction Regulation, Shanghai Institute for Biomedical and Pharmaceutical Technologies, Shanghai 200237, China; ^5^Shanghai Key Laboratory of Birth Defects, Children's Hospital of Fudan University, Shanghai, China

**Keywords:** cohort study, digit ratio, prenatal depression

## Abstract

Animal studies have indicated that prenatal depression may affect the reproductive development of offspring. The digit ratio has been proposed as a marker of in utero reproductive development. The aim of this study was to explore the association between prenatal depression and the digit ratio (2nd:4th digit ratio (2D:4D)) in children. This study involved 668 mother–child pairs enrolled in the Shanghai–Minhang birth cohort study (S–MBCS). Prenatal depressive symptoms among pregnant women were evaluated during recruitment and late pregnancy using the validated Chinese version of the Center for Epidemiological Studies Depression Scale (CES-D). Measurements of digit lengths of both hands were conducted during follow-up visits at 4 and 6 years. We observed that mothers with prenatal depressive symptoms tended to have offspring with higher digit ratios at 4 and 6 years of age. For children whose mothers experienced depressive symptoms in the second trimester, the digit ratio of the left hand (2D:4DL) at 4 years of age increased by 0.007 (95% CI: 0.000, 0.015) in the subthreshold group and 0.010 (95% CI: 0.001, 0.019) in the screen-positive group. For those with depressive symptoms in the third trimester, the 2D:4DL in the screen-positive group increased by 0.012 (95% CI: 0.001, 0.023) at 4 years of age and 0.014 (95% CI: 0.003, 0.024) at 6 years of age. A dose–response relationship was established for both the strength and duration of depressive symptoms. Our study suggests that prenatal depressive symptoms may perturb the reproductive development of offspring and predominantly exhibit a feminizing effect.

## 1. Introduction

Prenatal depression is a psychological disorder that affects more than 20% of pregnant women worldwide [1, 2], with a higher prevalence in developing countries [3]. Despite its high prevalence, prenatal depression has received much less attention relative to the research focused on postpartum depression. Studies have indicated that prenatal depression, even at levels that do not meet clinical diagnostic criteria, can increase the risk of complications during pregnancy, including preeclampsia and placental abruption [4, 5].

In addition to the adverse effects of prenatal depression on mothers, greater concerns may be related to the fetus. Given that the intrauterine developmental stage is a critical period for fetal growth and is characterized by high vulnerability, disruptions during this period can result in deviations from the normal developmental trajectory. Accumulating human evidence suggests that prenatal depression may lead to alterations in the intrauterine environment and produce long-term health effects in offspring [6, 7]. Notably, depression is associated with changes in the hormone profiles of women, such as altered plasma estradiol and testosterone levels [8, 9]. This raises concerns regarding its potential impact on reproductive function, which is the most sensitive target of endogenous hormone homeostasis. However, there is currently no evidence demonstrating the effects of prenatal depression on the reproductive development of offspring in humans. Animal studies have shown shortened mounting or sex behavior latency, changes in estrous cycle length, and reduced anogenital distance (AGD) in offspring resulting from prenatal stress exposure, which is a widely used strategy for the establishment of animal models of depression. This suggests that prenatal stress may disrupt reproductive development, as reported in two other studies [10, 11].

A significant challenge in exploring the effects of prenatal exposure on offspring reproductive outcomes in epidemiological studies is the long period required for monitoring, as it takes many years for the offspring to reach sexual maturity. The 2nd:4th digit ratio (2D:4D), a noninvasive marker that has been validated to be sensitive to hormonal disturbances in utero [12], has been increasingly used in human studies to assess the association between prenatal adverse events and reproductive development in offspring [13, 14]. Previous studies suggested that the 2D:4D ratio is determined by the balance between prenatal testosterone and estrogen levels. A lower 2D:4D ratio indicates high fetal testosterone and low estrogen exposure, which are associated with a more masculine effect. Conversely, a higher 2D:4D ratio indicates a feminine effect [15]. Moreover, the 2D:4D ratio has been shown to be associated with reproductive problems that emerge later in life, including genital abnormalities, reduced sperm count, premature ejaculation, and abnormal circulating estradiol levels [16–19], making it an ideal early marker for the evaluation of long-term reproductive development alterations.

We hypothesized that prenatal depressive symptoms, owing to their effects on prenatal sex hormones, may influence reproductive development. This study aimed to test this hypothesis by examining the association between prenatal depressive symptoms and digit ratio in children aged 4 and 6 years in a large population-based longitudinal cohort study in Shanghai, China. Additionally, the 2D:4D ratio is a sexually dimorphic trait, with males typically exhibiting lower ratios than females [20]. A previous study found that the 2D:4D ratio in the left hand was negatively associated with amniotic fluid testosterone levels in female offspring [21]. While another study reported a significant negative association between the 2D:4D ratio in the right hand and the fetal testosterone or estradiol ratio [22]. Hence, we further explored the differences in the 2D:4D ratio between the left and right hands, including between male and female offspring exposed to maternal prenatal depressive symptoms.

## 2. Methods

### 2.1. Study Participants and Design

This study was based on the Shanghai–Minhang birth cohort study (S–MBCS), which is an ongoing prospective cohort study. Pregnant women at 12–16 weeks of gestation were recruited during their first prenatal examination between April and December 2012 at the Minhang Maternal and Child Health Hospital in Shanghai, China. Women were eligible for the study if they: (1) were native Chinese and residents of Shanghai, (2) intended to deliver at the study hospital, (3) had no history of major chronic diseases, and (4) were willing to attend scheduled interviews during pregnancy and after delivery.

A total of 1292 pregnant women were recruited, of whom 67 were excluded due to delivery at another hospital (*n* = 28), abortion or stillbirth (*n* = 31), or twin pregnancy (*n* = 8), and leaving 1225 live singletons delivered in the study hospital. Detailed information on the sociodemographic characteristics, health conditions, depressive symptoms, and reproductive histories of the participants was collected using structured questionnaires during recruitment (12–16 gestational weeks) and follow-up visits during late pregnancy (32–36 gestational weeks). At the 4 and 6-years follow-up visits, the 2D:4D ratio and other anthropometric indices of 603 and 540 children, respectively, were measured. A total of 668 mother–child pairs with information on prenatal depressive symptoms and at least one 2D:4D measurement were included in this study.

### 2.2. Prenatal Depressive Symptoms Assessment

Prenatal depressive symptoms in pregnant women were evaluated during recruitment and in late pregnancy using the validated Chinese version of the Center for Epidemiological Studies Depression Scale (CES-D), a widely used self-assessment scale for depression screening. The CES-D consists of 20 items and assigns a score ranging from 0 to 3 to each item, with the sum of all items calculated as the total score. It quantifies depressive symptoms from several perspectives, including depressed mood, feelings of guilt, worthlessness, helplessness, hopelessness, energy loss, and sleep and appetite disturbances. Higher CES-D scores indicated more severe depressive symptoms. Previous studies have highlighted the adverse effects of subthreshold depressive symptoms [23]. Therefore, to provide a comprehensive understanding of the impact of depressive symptoms during pregnancy, participants were categorized into three groups: the screen-positive depressive symptoms group (with a total CES-D score of 16 or above) [24], the subthreshold depressive symptoms group (with scores ranging from 10 to 15) [25], and the nondepressive symptoms group (with scores ranging from 0 to 9). The internal consistency of the CES-D scale in our cohort was acceptable (Cronbach's *α* = 0.68 in the second trimester, 0.72 in the third trimester).

### 2.3. 2D:4D Measurements

Measurements of digit length in both hands were conducted by trained technicians during follow-up visits at ages 4 and 6 years. Vernier calipers (Scienceware, SP Bel-Art, USA) were used to measure the lengths of the second and fourth fingers at a resolution of 0.1 mm, from the midpoint of the crease at the base of the finger (the most proximal to the wrist) to the fingertip when the fingers were stretched. The examiners were blinded to the children's information. At the 4-year visit, one measurement was performed for each finger length, whereas at the 6-year visit, all measurements were repeated twice and the average of the two measurements was calculated. In addition, the finger lengths of nine children were measured a second time by an independent examiner during the 6-year follow-up visit. Intraclass correlation coefficients (ICCs) were calculated to assess intra- and interexaminer reliability across repeated digit ratio measurements. Previously, detailed information regarding ICCs has been provided, demonstrating moderate-to-good reliability of digit ratio measurements [14].

### 2.4. Statistical Analysis

Chi-squared tests and *t*-tests were conducted to compare the demographic characteristics of the included and excluded mother–child pairs. The distribution of maternal CES-D scores measured in the second and third trimesters and the 2D:4D scores of children aged 4 and 6 years were tabulated. Generalized linear models were used to assess the associations between prenatal depressive symptoms in the second and third trimesters of pregnancy and 2D:4D ratios in children aged 4 and 6 years, respectively. The nondepressive symptom group was used as the reference. Considering the sexually dimorphic nature of 2D:4D [15], we evaluated the interactions between sex and prenatal depressive symptoms using an *α* level of 0.10, since it was considered as an acceptable significance level in exploratory analysis [26] and found significant interactions for most comparisons; thus, stratified analyses by sex were conducted. To explore the effect of the duration of prenatal depressive symptoms on 2D:4D ratios, participants in the screen-positive depressive symptoms group in both trimesters were assigned to the persistent depressive symptoms group. In contrast, those in the nondepressive symptoms group in both trimesters were assigned to the persistent nondepressive symptoms group. The remaining participants were assigned to the brief depressive symptoms group [27]. The associations of different depressive level groups or duration groups with 2D:4D ratios were assessed for linear trends using categorical exposure variables as continuous parameters. Furthermore, a generalized estimating equation (GEE) was used to assess the association between prenatal depressive symptoms and digit ratios, considering the correlation between measurements at the ages of 4 and 6 years.

Covariates were selected based on previous literature or were included in the models if they altered the coefficients by >10%. The following covariates were adjusted for in all models: gestational age (weeks), maternal age at delivery (years), paternal age at delivery (years), parity (1 and ≥2), household income per capita (<4000, 4000–8000, and >8000 RMB/month), maternal education level (high school or below, undergraduate degree or associate degree, and postgraduate degree or above), maternal prepregnancy body mass index (BMI; <18.5, 18.5–24, and ≥24 kg/m^2^), maternal prepregnancy passive smoking (yes or no), paternal prepregnancy alcohol consumption (yes or no), and feeding patterns during the first 6 months (exclusively breastfed, mixed, and exclusively formula fed). The adjusted models were checked for collinearity using the variance inflation factors for each variable.

We repeated the main analysis in children born full-term to eliminate the potential effect of preterm birth, since previous studies have reported an association between prenatal depression and preterm birth [28] and preterm birth was also found to be a risk factor for impaired offspring development [29].

All statistical analyses were performed using the SAS 9.4 (SAS Institute Inc., Cary, NC, USA). All the abovementioned statistical analyses were two-sided, with *p*-value <0.05 as statistically significant.

### 2.5. Ethics Statement

All pregnant women provided written informed consent for themselves and their children at recruitment and each subsequent visit. The cohort protocols were approved by the Ethics Committee of the Shanghai Institute of Biomedical and Pharmaceutical Technologies.

## 3. Results

### 3.1. Distribution of Participants' Characteristics

The mean age and gestational age at delivery in the 668 pregnant women included in this study were 28.6 years and 39.5 weeks, respectively. Most pregnant women included in the study were nulliparous (86.3%). Additionally, a significant proportion of women (79.3%) attained a college-level education or higher, and with a prepregnancy BMI within the normal range (73.3%). Approximately 40% of the pregnant women reported passive smoking before pregnancy and nearly 32% reported that their spouses consumed alcohol before pregnancy. The characteristics of the included and excluded mother–child pairs were comparable, except that the included mothers tended to have higher education levels (college and above: 79.3% vs. 71.6%; Table 1).

### 3.2. Distribution of Prenatal Depressive Symptoms and 2D:4D Ratios in Children

Table 1 demonstrates that there was no significant difference in prenatal depression status between the pregnant women who were included and those who were excluded from the study. Among the included pregnant women, the proportions of women in the screen-positive depressive symptom and subthreshold depressive symptom groups at recruitment (second trimester) were 19.8% and 34.6%, respectively. The CES-D scores between the second and third trimesters were moderately correlated (Pearson's correlation coefficient, 0.40; *p* < 0.0001). In the second trimester, 19.8% of women experienced screen-positive depressive symptoms, while 9.0% experienced screen-positive depressive symptoms in the third trimester. Similarly, the percentages of women experienced subthreshold depressive symptoms in the second and third trimesters were 34.6% and 22.6%, respectively.

The distribution of the 2D:4D ratios for both hands in children aged 4 and 6 years is presented in Table 1. The average 2D:4D ratio in the left hand was higher than that in the right hand and boys generally had lower 2D:4D ratios than girls; however, this difference was not statistically significant. The 2D:4D ratios measured at 4 and 6 years of age were moderately correlated (Pearson's correlation coefficients ranging from 0.37 to 0.63, *p* < 0.0001).

### 3.3. Association Between Prenatal Depressive Symptoms and 2D:4D Ratios in Children

Table 2 demonstrates a general pattern that prenatal depressive symptoms were associated with higher 2D:4D ratios in the offspring. Specifically, for depressive symptoms in the second trimester, a statistically significant increase in digit ratios was observed in the left hand of the offspring (2D:4DL) at 4 years of age, with increments of 0.007 (95% CI: 0.000, 0.015) for the subthreshold depressive group and 0.010 (95% CI: 0.001, 0.019) for the screen-positive depressive group. In relation to the depressive symptoms in the third trimester, associations were observed on 2D:4DL of offspring at both 4 and 6 years of age, although statistically significant increases were only found in the screen-positive depressive group (4-years: *β*: 0.012, 95% CI: 0.001, 0.023 and 6-years: *β*: 0.014, 95% CI: 0.003, 0.024). A dose–response effect was observed across different levels of the depressive group in all the above associations. Stratification by sex showed a similar pattern, with an additional association with an increased 2D:4DR ratio among girls aged 6 years in the third trimester (Table 2).

Children of mothers who had persistent depressive symptoms exhibited the highest 2D:4DL ratio at the age of 4 years compared to those whose mothers had experienced brief or nondepressive symptoms. Furthermore, a significant dose–response relationship was observed. However, no such associations were observed in other comparisons (Table 3).

The GEE models examined the association between prenatal depressive symptoms and repeated measures of the digit ratio in the offspring (Table 4). We observed increased 2D:4DL in both subthreshold and screen-positive groups at second trimester (subthreshold group: *β*: 0.007, 95% CI: 0.000, 0.013 and screen-positive group: *β*: 0.009, 95% CI: 0.002, 0.018) and third trimester (subthreshold group: *β*: 0.007, 95% CI: 0.000, 0.015 and screen-positive group: *β*: 0.014, 95% CI: 0.005, 0.023). When stratified by sex, similar results were found with additional associations between increased 2D:4DR among boys (in the second-trimester subthreshold group) and girls (in the third-trimester screen-positive group). Furthermore, as expected, the offspring of mothers with persistent prenatal depressive symptoms had the highest 2D:4DL ratios among the offspring in the GEE model. However, after stratification by sex, a significant association of the increased 2D:4DL ratio only remained among girls. Moreover, an additional association with increased 2D:4DR ratio was observed among girls in the persistent depressive group (Table 5).

Patterns consistent with the main analyses were found when we restricted the analyses to mother–child pairs in full-term offspring (Table S1).

## 4. Discussion

In this longitudinal birth cohort study, mothers with prenatal depressive symptoms tended to have offspring with higher digit ratios at 4 and 6 years of age. Dose–response relationships were observed, with offspring whose mothers had higher levels or longer durations of prenatal depressive symptoms tending to have higher digit ratios at 4 and 6 years of age.

To the best of our knowledge, our study provides the first epidemiological evidence assessing the effect of prenatal depressive symptoms on reproductive development in offspring and suggests a feminizing effect in both sexes. Several animal studies have shown that prenatal stress, commonly used as an alternative in rodent models of depressive symptoms, is associated with reduced copulatory behavior [30] and AGD in male offspring [11], suggesting a potential feminizing effect of prenatal stress or depressive symptoms, which is consistent with our findings. Only one human study has investigated the association between prenatal exposure to stressful life events and AGD in offspring, yielding results consistent with our findings in male infants, in which a trend towards shorter AGDs was observed, but among female infants, longer AGDs were observed [31]. The classification of prenatal stress in that study was based on personal experiences rather than depressive symptoms, making it less comparable to our study. Further studies are needed to verify the relationship between prenatal depressive symptoms and reproductive development in the offspring.

The mechanisms underlying the effects of prenatal depressive symptoms on the offspring digit ratio are unknown. Several animal studies have reported reduced testosterone concentrations in newborn and adult offspring exposed to prenatal stress [32–34]. Stress, as well as depression it may induce, is widely observed to induce hormone release from the hypothalamic–pituitary–adrenal (HPA) axis, including corticosterone and *β*-endorphin, which can cross the placental barrier [35, 36] and may further affect the release and production of testosterone. Therefore, the interaction between the HPA and hypothalamic–pituitary–gonadal axes may result in decreased testosterone production in fetuses exposed to prenatal stress, leading to a feminization effect on the reproductive development of the offspring.

The sex-specific effect of prenatal depression on children's reproductive development, as reported in this study, is more pronounced in girls and is supported by human evidence. Gaml-Sørensen et al. [37] reported that prenatal exposure to maternal life stress and emotional distress during pregnancy was associated with altered pubertal timing in offspring, particularly in girls. One biological explanation was that maternal stress during pregnancy might influence the prenatal hormonal environment in a sex-dependent manner, especially for testosterone, and that in utero hormone homeostasis is vital for the differentiation of sexually dimorphic areas involved in reproductive development [38]. Furthermore, previous studies have found a more pronounced negative association between the 2D:4D ratio and amniotic testosterone in girls [21, 39], suggesting that girls may be more susceptible to in utero hormone changes than boys, although this fetal programming effect needs to be further investigated.

In the present study, increased digit ratios in offspring were observed not only in the screen-positive depressive symptom group but also in the subthreshold group. This was partly supported by previous studies that highlighted the adverse effects of prenatal subthreshold depressive symptoms, which were associated with an increased risk of emotional and behavioral problems in the offspring [40, 41]. Our study contributes to the accumulating evidence that subthreshold depressive symptoms, although not meeting the diagnostic criteria for depression, may also induce health risks.

We observed a stronger association between a longer duration of prenatal depressive symptoms and an increased digit ratio in offspring. Consistent findings have been observed in previous studies that reported that persistent depressive symptoms were stronger risk factors for developmental delay, cognitive impairment, and social–emotional problems than brief depression in offspring [27, 42]. Our study highlights the need for increased attention to pregnant women with persistent depressive symptoms [38].

This study has several strengths. First, the prospective design of the S–MBCS cohort guaranteed a temporal relationship between prenatal depressive symptoms and the digit ratio in offspring. Second, repeated measurements of digit ratio during childhood provided more reliable evidence of the longitudinal effects of prenatal exposure on depressive symptoms. Despite its strengths, this study has several limitations. First, some participants in the original cohort were not included because they were lost to follow-up, although the included mothers generally had characteristics similar to those excluded. Lost-to-follow-up is one of the most common problems in longitudinal studies that require long-term follow-up. In the present study, the loss of participants to follow-up was mostly due to relocation or changes in contact information, which are less likely to be related to exposure and outcomes. Thus, loss is not expected to lead to a substantial bias. Second, previous evidence indicated an association between antidepressant medication use during pregnancy and adverse neonatal outcomes [43]. Information on the treatment for depression during pregnancy was not collected from the participants, which may have led to residual confounding. However, our findings showed a consistent association between increased digit ratios in offspring among both the screen-positive and subthreshold groups for prenatal depressive symptoms and that women in the subthreshold group were not expected to use any medication. Therefore, the confounding effects of the medications may not have significantly affected the observed association. Third, although the assessment of intra- and interexaminer ICCs based on repeated 2D:4D measurements at the 6-year follow-up visit showed moderate to good reliability, the precision of the 2D:4D ratio measurements at 4 years of age is a cause for concern because the length of each finger was measured only once. However, this may produce a nondifferential misclassification and bias the results towards the null hypothesis.

## 5. Conclusions

In this prospective cohort study, we observed that prenatal depressive symptoms were associated with an increased 2D:4D digit ratio in offspring of both sexes at 4 and 6 years of age. Dose–response relationships were found with regard to both the strength and duration of depressive symptoms. Our study suggests that prenatal depressive symptoms may perturb the reproductive development of offspring and predominantly exhibit a feminizing effect. Our findings advocate an increased focus on screening and intervention for prenatal depressive symptoms to potentially mitigate the long-term effects on offspring reproductive health.

## Figures and Tables

**Table 1:** Characteristics of included and excluded mother–child pairs.

Characteristics	Mother–child pairs included in the analysis			Mother–child pairs excluded in the analysis (*n* = 557)
	Total (*n* = 668)	Boy (*n* = 370)	Girl (*n* = 280)	
Maternal age at delivery				
<25	75 (11.4)	51 (13.8)*⁣*^*∗*^	23 (8.2)	70 (12.9)
25–29	348 (52.8)	199 (53.8)	145 (51.8)	300 (55.2)
30–34	200 (30.3)	99 (26.8)	98 (35.0)	152 (27.9)
≥35	36 (5.5)	21 (5.6)	14 (5.0)	22 (4.0)
Paternal age at delivery				
<30	296 (45.2)	176 (47.9)	118 (42.3)	241 (44.9)
25–39	328 (50.1)	172 (46.9)	149 (53.4)	277 (51.6)
≥40	31 (4.7)	19 (5.2)	12 (4.3)	19 (3.5)
Gestational age at delivery				
<37	24 (3.6)	10 (2.7)	12 (4.3)	22 (3.9)
≥37	644 (96.4)	360 (97.3)	268 (95.7)	535 (96.1)
Maternal prepregnancy BMI (kg/m^2^)				
<18.5	119 (18.2)	72 (19.8)	45 (16.4)	125 (22.8)
18.5–24	481 (73.3)	257 (70.6)	209 (76.3)	381 (69.5)
≥24	56 (8.5)	35 (9.6)	20 (7.3)	42 (7.7)
Parity				
1	571 (86.3)	309 (84.4)	248 (89.2)	457 (82.5)
≥2	91 (13.7)	57 (15.6)	30 (10.8)	97 (17.5)
Maternal prepregnancy passive smoking				
Yes	272 (40.8)	154 (41.7)	111 (39.8)	222 (40.0)
No	394 (59.2)	215 (58.3)	168 (60.2)	333 (60.0)
Paternal prepregnancy alcohol consumption				
Yes	213 (31.9)	127 (34.3)	80 (28.7)	180 (32.6)
No	454 (68.1)	243 (65.7)	199 (71.3)	372 (67.4)
Child sex				
Boy	370 (56.9)	—	—	278 (52.7)
Girl	280 (43.1)	—	—	250 (47.3)
Household income per capita (RMB/month)				
<¥ 4000	128 (19.4)	80 (21.8)	45 (16.4)	125 (22.8)
¥ 4000–8000	265 (40.1)	147 (40.0)	112 (40.7)	224 (40.8)
>¥ 8000	267 (40.5)	140 (38.2)	118 (42.9)	200 (37.4)
Maternal education				
High school or below	138 (20.7)*⁣*^*∗*^	81 (21.9)	53 (18.9)	158 (28.4)
Undergraduate degree/Associate degree	460 (68.9)	254 (68.6)	195 (69.7)	370 (66.4)
Postgraduate degree or above	70 (10.4)	35 (9.5)	32 (11.4)	29 (5.2)
Feeding patterns during the first 6 months				
Exclusively breastfed	311 (47.3)	169 (46.1)	131 (47.8)	151 (44.5)
Mixed	301 (45.7)	172 (46.9)	124 (45.3)	154 (45.4)
Exclusively formula fed	46 (7.0)	26 (7.0)	19 (6.9)	34 (10.1)
Maternal depressive status in the 2^nd^ trimester				
Nondepressive symptoms (≤9)	305 (45.6)	174 (47.0)	124 (44.3)	232 (41.7)
Subthreshold depressive symptoms (10–15)	231 (34.6)	125 (33.8)	100 (35.7)	206 (36.9)
Screen-positive depressive symptoms (≥16)	132 (19.8)	71 (19.2)	56 (20.0)	119 (21.4)
Maternal depressive status in the 3^rd^ trimester				
Nondepressive symptoms (≤9)	457 (68.4)	255 (68.9)	186 (66.4)	396 (71.1)
Subthreshold depressive symptoms (10–15)	151 (22.6)	79 (21.4)	72 (25.7)	116 (20.8)
Screen-positive depressive symptoms (≥16)	60 (9.0)	36 (9.7)	22 (7.9)	45 (8.1)
2D:4DL aged 4^a^	0.959 (0.037)	0.957 (0.036)	0.962 (0.039)	—
2D:4DR aged 4^a^	0.955 (0.036)	0.954 (0.037)	0.956 (0.035)	—
2D:4DL aged 6^a^	0.968 (0.034)	0.966 (0.036)	0.969 (0.032)	—
2D:4DR aged 6^a^	0.965 (0.031)	0.963 (0.030)	0.966 (0.033)	—

*Note:* Missing values of mother–child pairs included in the analysis: Maternal age at delivery (*n* = 9), paternal age at delivery (*n* = 13), maternal prepregnancy BMI (*n* = 12), parity (*n* = 6), maternal prepregnancy passive smoking (*n* = 2), paternal prepregnancy alcohol consumption (*n* = 1), child sex (*n* = 18), household income per capita (*n* = 8), feeding patterns during the first 6 months (*n* = 10); 2D:4DL at aged 4 (*n* = 65), 2D:4DR at aged 4 (*n* = 66), 2D:4DL at aged 6 (*n* = 128), and 2D:4DR at aged 6 (*n* = 128). 2D:4DL, digit ratios of the left hand; 2D:4DR, digit ratios of the right hand.

^a^Mean (SD).

*⁣* ^*∗*^Statistically significant differences (*p* < 0.05) using Chi-squared test.

**Table 2:** Associations between prenatal depressive symptoms and digit ratios in children aged 4 and 6 years^a^.

Group	Depressive symptoms in the 2^nd^ trimester			Depressive symptoms in the 3^rd^ trimester		
	*β* (95% CI)			*β* (95% CI)		
	Total	Boy	Girl	Total	Boy	Girl
2D:4DL aged 4 years						
Nondepressive group	Ref^#^	Ref^#^	Ref	Ref^#^	Ref	Ref^#^
Subthreshold group	0.007 (0.000, 0.015)*⁣*^*∗*^	0.013 (0.004, 0.022)*⁣*^*∗*^	0.002 (−0.011, 0.014)	0.005 (−0.003, 0.013)	−0.001 (−0.012, 0.009)	0.013 (0.001, 0.026)*⁣*^*∗*^
Screen-positive group	0.010 (0.001, 0.019)*⁣*^*∗*^	0.009 (−0.002, 0.021)	0.011 (−0.004, 0.026)	0.012 (0.001, 0.023)*⁣*^*∗*^	0.008 (−0.006, 0.023)	0.021 (0.002, 0.040)*⁣*^*∗*^
2D:4DR aged 4 years						
Nondepressive group	Ref	Ref	Ref	Ref	Ref	Ref
Subthreshold group	0.004 (−0.003, 0.011)	0.008 (−0.002, 0.018)	0.001 (−0.010, 0.012)	0.003 (−0.004, 0.012)	0.000 (−0.011, 0.011)	0.009 (−0.003, 0.019)
Screen-positive group	0.006 (−0.002, 0.015)	0.009 (−0.003, 0.021)	0.004 (−0.009, 0.017)	0.000 (−0.011, 0.011)	−0.005 (−0.019, 0.010)	0.008 (−0.008, 0.025)
2D:4DL aged 6 years						
Nondepressive group	Ref	Ref	Ref	Ref^#^	Ref	Ref^#^
Subthreshold group	0.003 (−0.004, 0.010)	0.008 (−0.001, 0.018)	−0.004 (−0.015, 0.006)	0.005 (−0.003, 0.013)	0.002 (−0.009, 0.013)	0.007 (−0.004, 0.018)
Screen-positive group	0.007 (−0.002, 0.016)	0.003 (−0.009, 0.015)	0.010 (−0.003, 0.023)	0.014 (0.003, 0.024)*⁣*^*∗*^	0.011 (−0.003, 0.025)	0.019 (0.004, 0.034)*⁣*^*∗*^
2D:4DR aged 6 years						
Nondepressive group	Ref	Ref	Ref	Ref	Ref	Ref^#^
Subthreshold group	0.003 (−0.004, 0.009)	0.004 (−0.005, 0.012)	0.003 (−0.008, 0.014)	0.002 (−0.005, 0.009)	−0.003 (−0.013, 0.006)	0.008 (−0.004, 0.019)
Screen-positive group	0.002 (−0.006, 0.010)	−0.006 (−0.016, 0.005)	0.013 (−0.001, 0.027)	0.004 (−0.006, 0.014)	−0.004 (−0.017, 0.008)	0.017(0.000, 0.034)*⁣*^*∗*^

*Note*: 2D:4DL, digit ratios of the left hand; 2D:4DR, digit ratios of the right hand.

^a^Adjusted for gestational age at delivery, maternal age at delivery, paternal age at delivery, parity, household income per capita, maternal education, maternal prepregnancy body mass index, maternal prepregnancy passive smoking, paternal prepregnancy drinking, and feeding patterns during the first 6 months.

*⁣* ^*∗*^ *p* value < 0.05.

^#^ *p* trend < 0.05.

**Table 3:** Associations between duration of prenatal depressive symptoms and digit ratios in children aged 4 and 6 years^a^.

Group		Duration of prenatal depressive symptoms				
		*β* (95% CI)				
	*n*	Total	*n*	Boy	*n*	Girl
2D:4DL aged 4 years						
Persistent nondepressive group	219	Ref^#^	131	Ref^#^	85	Ref^#^
Brief depressive group	308	0.009 (0.003, 0.016)*⁣*^*∗*^	169	0.011 (0.002, 0.019)*⁣*^*∗*^	133	0.010 (−0.002, 0.022)
Persistent depressive group	30	0.016 (0.001, 0.031)*⁣*^*∗*^	18	0.007 (−0.011, 0.026)	12	0.029 (0.005, 0.055)*⁣*^*∗*^
2D:4DR aged 4 years						
Persistent nondepressive group	219	Ref	131	Ref	85	Ref
Brief depressive group	307	0.007 (0.000, 0.013)*⁣*^*∗*^	168	0.009 (−0.000, 0.017)	133	0.006 (−0.004, 0.017)
Persistent depressive group	30	0.005 (−0.009, 0.019)	18	−0.004 (−0.023, 0.016)	12	0.018 (−0.004, 0.039)
2D:4DL aged 6 years						
Persistent nondepressive group	206	Ref	126	Ref	77	Ref
Brief depressive group	266	0.006 (−0.001, 0.013)	149	0.006 (−0.003, 0.015)	115	0.003 (−0.006, 0.013)
Persistent depressive group	30	0.007 (−0.007, 0.021)	19	−0.002 (−0.019, 0.016)	11	0.019 (−0.001, 0.039)
2D:4DR aged 6 years						
Persistent nondepressive group	206	Ref	126	Ref	77	Ref
Brief depressive group	266	0.003 (−0.003, 0.009)	149	−0.001 (−0.009, 0.007)	115	0.009 (−0.002, 0.019)
Persistent depressive group	30	−0.000 (−0.013, 0.013)	19	−0.009 (−0.025, 0.007)	11	0.015 (−0.008, 0.037)

^a^Adjusted for gestational age at delivery, maternal age at delivery, paternal age at delivery, parity, household income per capita, maternal education, maternal prepregnancy body mass index, maternal prepregnancy passive smoking, paternal prepregnancy drinking, and feeding patterns.

*⁣* ^*∗*^ *p* value < 0.05.

^#^ *p* trend < 0.05.

**Table 4:** Associations between prenatal depressive symptoms and digit ratios in children in GEE model^a^.

Group	Depressive symptoms in the 2^nd^ trimester			Depressive symptoms in the 3^rd^ trimester		
	*β* (95% CI)			*β* (95% CI)		
	Total	Boy	Girl	Total	Boy	Girl
2D:4DL						
Nondepressive group	Ref	Ref	Ref	Ref	Ref	Ref
Subthreshold group	0.007 (0.000, 0.013)*⁣*^*∗*^	0.016 (0.008, 0.024)*⁣*^*∗*^	−0.003 (−0.013, 0.007)	0.007 (0.000, 0.015)*⁣*^*∗*^	0.005 (−0.005, 0.015)	0.011 (−0.000, 0.021)
Screen-positive group	0.009 (0.002, 0.018)*⁣*^*∗*^	0.008 (−0.002, 0.018)	0.012 (−0.001, 0.025)	0.014 (0.005, 0.023)*⁣*^*∗*^	0.011 (−0.002, 0.024)	0.021 (0.007, 0.036)*⁣*^*∗*^
2D:4DR						
Nondepressive group	Ref	Ref	Ref	Ref	Ref	Ref
Subthreshold group	0.004 (−0.002, 0.009)	0.009 (0.001, 0.016)*⁣*^*∗*^	−0.001 (−0.010, 0.009)	0.004 (−0.003, 0.009)	−0.000 (−0.008, 0.008)	0.008 (−0.001, 0.018)
Screen-positive group	0.004 (−0.004, 0.011)	0.001 (−0.008, 0.011)	0.007 (−0.004, 0.019)	0.001 (−0.007, 0.009)	−0.006 (−0.017, 0.004)	0.012 (0.001, 0.024)*⁣*^*∗*^

^a^Adjusted for gestational age at delivery, maternal age at delivery, paternal age at delivery, parity, household income per capita, maternal education, maternal prepregnancy body mass index, maternal prepregnancy passive smoking, paternal prepregnancy drinking, and feeding patterns.

*⁣* ^*∗*^ *p* < 0.05.

**Table 5:** Associations between duration of prenatal depressive symptoms and digit ratios in children in GEE model^a^.

Group	Duration of prenatal depressive symptoms		
	*β* (95% CI)		
	Total	Boy	Girl
2D:4DL			
Persistent nondepressive group	Ref	Ref	Ref
Brief depressive group	0.009 (0.004, 0.016)*⁣*^*∗*^	0.013 (0.006, 0.021)*⁣*^*∗*^	0.007 (−0.002, 0.017)
Persistent depressive group	0.014 (0.002, 0.026)*⁣*^*∗*^	0.006 (−0.009, 0.020)	0.027 (0.009, 0.046)*⁣*^*∗*^
2D:4DR			
Persistent nondepressive group	Ref	Ref	Ref
Brief depressive group	0.005 (−0.000, 0.011)	0.006 (−0.001, 0.013)	0.006 (−0.003, 0.015)
Persistent depressive group	0.003 (−0.008, 0.013)	−0.005 (−0.018, 0.008)	0.016 (0.002, 0.031)*⁣*^*∗*^

^a^Adjusted for gestational age at delivery, maternal age at delivery, paternal age at delivery, parity, household income per capita, maternal education, maternal prepregnancy body mass index, maternal prepregnancy passive smoking, paternal prepregnancy drinking, and feeding patterns.

*⁣* ^*∗*^ *p* < 0.05.

## Data Availability

The data that support the findings of this study are available on request from the corresponding author. The data are not publicly available due to privacy or ethical restrictions.

## Nomenclature

2D:4D: Second to fourth digit ratio
S–MBCS: Shanghai–Minhang birth cohort study
CES-D: Center for Epidemiological Studies Depression Scale
2D:4DL: Digit ratio of the left hand
2D:4DR: Digit ratio of the right hand
AGD: Anogenital distance
ICCs: Intraclass correlation coefficients
GEE: Generalized estimating equation
BMI: Body mass index
HPA: Hypothalamic–pituitary–adrenal.

## Supporting Information

Supporting Information Table S1. Associations between prenatal depressive symptoms and digit ratio in children aged 4 and 6 years in full-term group. We repeated the main analysis on full-term children to eliminate the potential effects of preterm birth. Regression coefficients (*β*s) and 95% confidence intervals (CIs) for the associations were estimated. The models were adjusted for gestational age at delivery, maternal age at delivery, paternal age at delivery, parity, household income per capita, maternal education, maternal prepregnancy body mass index, maternal prepregnancy passive smoking, paternal prepregnancy drinking, and feeding patterns. Table S2. The *p*-value for the interaction effects of prenatal depressive symptoms and sex on digit ratios. The *p* value for the interaction effects of prenatal depressive symptoms and sex on digit ratios were calculated. Models were adjusted for gestational age at delivery, maternal age at delivery, household income per capita, maternal prepregnancy body mass index, maternal prepregnancy passive smoking, and feeding patterns during the first 6 months.
