# Supplementary material for: Association of Prenatal Depression With Second to Fourth Digit Ratio in Children Aged 4 and 6 Years
Source: Depress Anxiety. 2025 May 26;2025:6655082. doi: 10.1155/da/6655082 (PMC12129599; doi:10.1155/da/6655082)
Supplement: Supporting Information — Table S1. Associations between prenatal depressive symptoms and digit ratio in children aged 4 and 6 years in full-term group. We repeated the main analysis on full-term children to eliminate the potential effects of preterm birth. Regression coefficients (βs) and 95% confidence intervals (CIs) for the associations were estimated. The models were adjusted for gestational age at delivery, maternal age at delivery, paternal age at delivery, parity, household income per capita, maternal education, maternal prepregnancy body mass index, maternal prepregnancy passive smoking, paternal prepregnancy drinking, and feeding patterns. Table S2. The p-value for the interaction effects of prenatal depressive symptoms and sex on digit ratios. The p value for the interaction effects of prenatal depressive symptoms and sex on digit ratios were calculated. Models were adjusted for gestational age at delivery, maternal age at delivery, household income per capita, maternal prepregnancy body mass index, maternal prepregnancy passive smoking, and feeding patterns during the first 6 months. [file 6655082.f1.docx]

Table S1 Associations between prenatal depressive symptoms and digit ratio in children aged 4 and 6 in full-term group ^a^

|  | | Depressive symptoms in the 2^nd^ trimester  β (95% CI) | | | Depressive symptoms in the 3^rd^ trimester  β (95% CI) | | |
| --- | --- | --- | --- | --- | --- | --- | --- |
|  |  | Total | Boy | Girl | Total | Boy | Girl |
| 2D:4DL aged 4 | Non-depressive group | ref ^#^ | ref ^#^ | ref | ref | ref | ref ^#^ |
|  | Subthreshold group | **0.008(0.000, 0.015)^*^** | **0.012(0.002,0.021)^*^** | 0.003(-0.010,0.015) | 0.003(-0.005, 0.011) | -0.003(-0.014,0.007) | 0.012(-0.001,0.025) |
|  | Screen-positive group | **0.010(0.001, 0.019)^*^** | 0.009(-0.002,0.021) | 0.012(-0.004,0.027) | **0.012(0.000, 0.023)^*^** | 0.008(-0.007,0.022) | **0.021(0.001,0.039)^*^** |
| 2D:4DR aged 4 | Non-depressive group | ref | ref | ref | ref | ref | ref |
|  | Subthreshold group | 0.004(-0.003, 0.011) | 0.007(-0.003,0.017) | 0.001(-0.009,0.012) | 0.002(-0.005, 0.010) | -0.001(-0.012,0.010) | 0.009(-0.002,0.020) |
|  | Screen-positive group | 0.007(-0.002, 0.015) | 0.008(-0.004,0.021) | 0.004(-0.009,0.017) | -0.000(-0.011, 0.011) | -0.005(-0.020,0.010) | 0.008(-0.008,0.025) |
| 2D:4DL aged 6 | Non-depressive group | ref | ref | ref | ref ^#^ | ref | ref ^#^ |
|  | Subthreshold group | 0.004(-0.003, 0.011) | 0.008(-0.002,0.018) | -0.003(-0.013,0.008) | 0.003(-0.005, 0.011) | 0.001(-0.009,0.012) | 0.006(-0.005,0.018) |
|  | Screen-positive group | 0.008(-0.001, 0.016) | 0.003(-0.009,0.015) | 0.012(-0.001,0.025) | **0.013(0.003,0.024)^*^** | 0.011(-0.004,0.025) | **0.019(0.004,0.035)^*^** |
| 2D:4DR aged 6 | Non-depressive group | ref | ref | ref | ref | ref | ref ^#^ |
|  | Subthreshold group | 0.002(-0.004, 0.009) | 0.002(-0.006,0.011) | 0.004(-0.007,0.016) | 0.002(-0.006, 0.009) | -0.004(-0.014,0.006) | **0.009(-0.003,0.022)** |
|  | Screen-positive group | 0.002(-0.007, 0.010) | -0.006(-0.016,0.004) | 0.014(-0.000,0.028) | 0.003(-0.006, 0.013) | -0.005(-0.017,0.008) | **0.017(0.001,0.034)^*^** |

^*^*p* value < 0.05；^#^*p* trend < 0.05

^a^ adjusted for gestational age at delivery, maternal age at delivery, paternal age at delivery, parity, household income per capita, maternal education, maternal pre-pregnancy body mass index, maternal pre-pregnancy passive smoking, paternal pre-pregnancy drinking, and feeding patterns.

Table S2 The *p-*value for the interaction effects of prenatal depressive symptoms and sex on digit ratios ^a^

|  | Depressive symptoms in the 2nd trimester (*p-*value) | Depressive symptoms in the 3rd trimester (*p*-value) |
| --- | --- | --- |
| 2D:4DL aged 4 | 0.164 | 0.085 |
| 2D:4DR aged 4 | 0.562 | 0.489 |
| 2D:4DL aged 6 | 0.038 | 0.525 |
| 2D:4DR aged 6 | 0.050 | 0.044 |

^a^ adjusted for gestational age at delivery, maternal age at delivery, household income per capita, maternal pre-pregnancy body mass index, maternal pre-pregnancy passive smoking, and feeding patterns during the first 6 months.
